# Ultrasound-guided Reduction of Colle’s fracture does not assist in Achieving Better Radiographic Indices - Results of a Randomised Controlled Trial

**DOI:** 10.5704/MOJ.2407.010

**Published:** 2024-07

**Authors:** MP Bhatt, SK Nema, M Ayyan

**Affiliations:** 1 Department of Orthopaedics, Jawaharlal Institute of Postgraduate Medical Education and Research (JIPMER), Puducherry, India; 2 Department of Orthopaedics, All India Institute of Medical Sciences (AIIMS), Raipur, India; 3 Department of Emergency Medicine, Jawaharlal Institute of Postgraduate Medical Education and Research (JIPMER), Puducherry, India

**Keywords:** distal radius fracture, Colle’s fracture, ultrasonography, manipulation, and reduction

## Abstract

**Introduction:**

Ultrasound-guided manipulation and reduction (M&R) of the distal radius fractures (DRF) is believed to improve radiographic indices due to real-time feedback of fracture alignment. The objective of this trial was to compare volar tilt, radial inclination, and radial height on radiographs between Ultrasound guided and conventional (landmark-guided) M&R.

**Materials and Methods:**

A total of 79 distal radius extraarticular fractures in adults were randomised to Ultrasound guided and conventional (landmark-guided) M&R. The radiograph parameters described above were compared before and after M&R in both groups.

**Results:**

Except for volar tilt (P=0.05 difference in difference), there was no difference in both the groups on radiograph parameters i.e. radial inclination and radial height. We estimated a reduction in the incidence of malreduction by 49% (Risk ratio 0.51) and an absolute risk reduction of 22% through USG-guided reduction. We evaluated a number needed to treat 4 through USG-directed M&R of DRF to prevent one unacceptable reduction. There were 9 (22%) and 18 (46%) (P=0.70) unacceptable reductions in USG-guided and landmark-guided M&R.

**Conclusion:**

Adding USG guidance to conventional landmark-based closed reduction methods is not beneficial for the accuracy of fracture reduction in Colle’s fracture. However, improved volar tilt in sonographic-directed M&R needs further studies to determine the clinical significance.

## Introduction

Distal radius fractures (DRF) account for one-sixth of fractures presented in the emergency rooms (ER)1. Closed reduction and plaster immobilisation is the most acceptable treatment for extra-articular DRF. However, radiographic guidance for manipulation and reduction (M&R) stay debated^1,2^. While radiographs estimate the accuracy of reduction at a point in time, fluoroscopy and ultrasonography offer a real-time assessment of the reduction of DRF. The demonstrated benefits of ultrasound use for DRF M&R include reduced ER waiting time and exposure to radiation^1^. However, using sonography and fluoroscopy to guide M&R is associated with a higher cost of treatment^2^. The diagnostic accuracy of Ultrasound for the identification of DRF and its efficacy in M&R in ER is well reported1. The use of both Portable machines as the point of care and standard units is reported^1,3^. However, these reports are cross-sectional, retrospective, case-control studies, and quasi-randomised controlled trials with heterogeneous participants (extra-articular and intra-articular DRF)^3,4^. The studies report the accuracy of Ultrasound against a set reference^1^.

The primary objective of this trial was to compare volar tilt between USG and conventional (blind landmark-based) M&R in DRF on radiographs. The secondary aim of this trial was to compare the radiograph indices of radial inclination and radial length in conventional (blind landmark-based) and Ultrasound-guided M&R in extra-articular DRF.

## Materials and Methods

This Open-label parallel-arm randomised controlled trial was conducted at a level III trauma centre from January 2020 to December 2021. The trial was registered with Clinical Trials Registry of India CTRI/2020/07/026934. Adult patients (>18 years of age) with less than five days old extra-articular fractures DRF: AO type 23 A 2.2 and 2.3 were randomised to two groups by permutative block randomisation of unequal blocks (sizes 4, 6, and 8).

Cases with open injuries, associated distal ulna fracture ipsilateral or contralateral upper extremity trauma interfering with evaluation were excluded from the study. Bilateral cases were also excluded from the study. The patients were enrolled in the study by the Senior Registrar of orthopaedics in the ER. Patients participated after Informed consent.

Group I (cases) comprised of DRF where Ultrasound directed manipulation and evaluation of the fracture reduction was done and maintained in a below elbow plaster of Paris cast. Group II (controls) comprised of DRF where the fracture site was manipulated without any imaging and maintained in a below elbow plaster of Paris cast.

The participants were randomised with the sequence number generation. Allocation concealment was done by SNOSE (sequentially numbered opaque sealed envelopes) as per the sequence generated by block randomisation. The random sequence generation and allocation concealment process was executed by orthopaedic senior residents who were not part of the study. The caregivers and the patients were not blinded for the treatment; however, the outcome assessors (radiologists) who estimated the primary and secondary outcomes on radiographs were blinded to the groups.

**Fig. 1:**
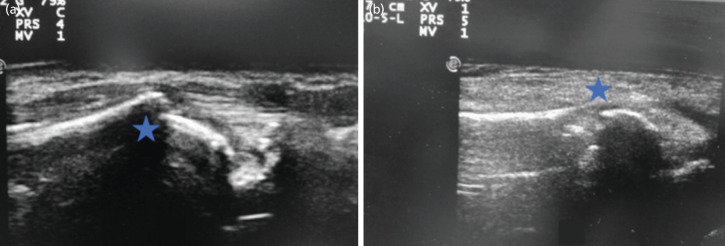
(a, b) Ultrasound before and after reduction in a case of distal radius extraarticular fracture.

An ultrasonography machine [Esaote MyLab50vision probe 7.5 to 10 MHz Genova Italy] estimated the sonographic assessments on closed reduction in group II. Real-time reviews of alignment on two orthogonal long-axis views (AP on the dorsal surface and lateral on the radial surface) on the Ultrasound along the distal radius assessed the adequacy of reduction (Fig. 1). An Emergency Medicine senior registrar evaluated the Ultrasound directed fracture reduction in ER. The Senior registrar was trained in ultrasound imaging for the distal radius in 15 normal forearms and then participated in the study.

DRF in both the groups was radiograph [Carestream DRX evolution the USA] (anteroposterior and lateral) before and after M&R. the Images were processed on picture archiving and communication software [GE electronics USA]. The cases were analysed irrespective of the reduction quality after the first manipulation in the group they were randomised. Both groups measured the primary and secondary outcomes before and after radiographs.

**Fig. 2:**
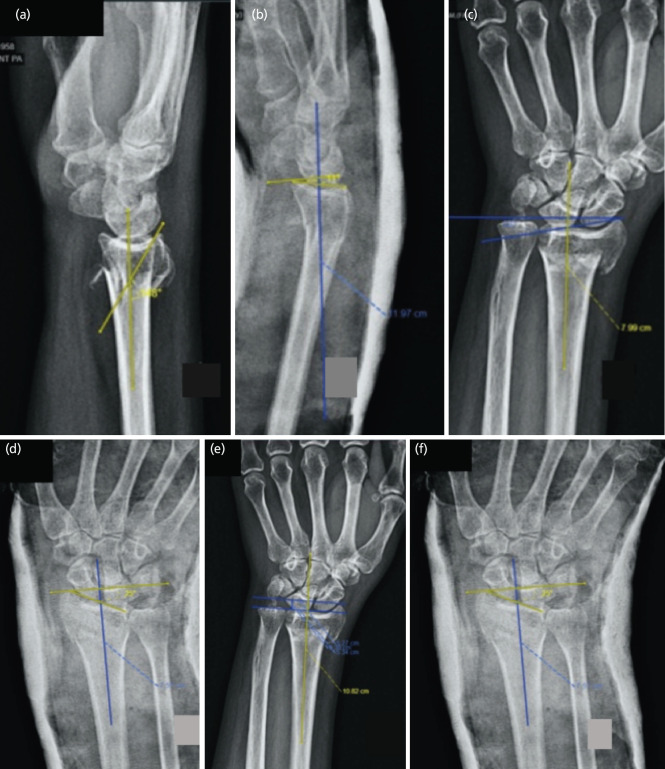
(a – f) Radiographic images, depicting method to measure volar tilt, radial inclination and radial height respectively in a case of distal radius extra-articular fracture.

The following radiographic parameters were the primary outcomes in both groups: (1) Volar tilt: the volar tilt of the distal radius was measured in degrees on a true lateral radiograph where the ulnar head was completely superimposed behind the radius. It was defined as the angle between a line drawn perpendicular to the long axis of the radius and a tangent line drawn along the slope of the dorsal-to-palmar surface of the radius (Fig. 2a, b). The values were assigned as positive and negative integers for volar and dorsal tilt before and after reduction radiographs. The mean with SD was estimated accordingly. (2) Radial inclination: the radial inclination, or radial angle, was measured in degrees on a PA radiograph between two lines; one line connecting the radial styloid tip and the ulnar aspect of the distal radius and a second line perpendicular to the longitudinal axis of the radius (Fig. 2c, d). The values were assigned as positive and negative integers for radial and ulnar inclination before and after reduction radiographs. The mean with SD was estimated accordingly. (3) Radial height: the distance between two lines drawn perpendicular to the long axis of the radius passing through the distal tip of the sigmoid notch at the distal ulnar articular surface of the radius and the distal end of the radial styloid on AP radiograph was defined as the radial height in millimetres (Fig. 2e, f).

A radial inclination of 15° to 25° (21° mean), the radial height of 5mm to 18mm (12mm mean), and a volar/palmar tilt of 0 to +22° (11° mean) were set as reference^5^. Deviation from any one or more of the set radiological parameters after M&R was defined as an unacceptable reduction. However, the endpoint to assess the primary and secondary outcome was fixed i.e. after first attempt, irrespective of the quality of reduction and whether or not second attempt is required.

Sample size, expecting a mean difference of 4 units and a pooled standard deviation of 6.5° in volar tilt between both the groups before and after manipulation, a sample size of 42 in each group was estimated, keeping a 5% level of confidence and 80% power in a ratio of 1:1 in both the groups^6^. Data was collected in a Microsoft Excel spreadsheet. Continuous variables were expressed as mean with standard deviation (SD), and categorical variables were expressed as frequency and percentages. Variables like age, volar tilt, radial inclination, radial height, etc., were compared between the groups by student t-test/Mann and Whitney U test. The data spread for normality was done using the Shapiro test. The radiographic outcomes were compared between the groups using paired t-test. Other variables like gender, fracture type, and mechanism of injury were compared using the Chi-squared test. Change in volar tilt, radial inclination, and radial length before and after the intervention was compared using the difference in difference analysis. A p-value of <0.05 was taken as significant. Analysis was carried out with STATA v 14.2.

## Results

We recruited 79 cases, randomised to either of the groups of 39 and 40 participants in groups I and II. More specifically, there were Five and Seven, 21 and nine, and seven and nine undisplaced (23 A2.1), dorsally displaced (23A2.2), and volar displaced fractures (23A2.3), respectively, in cases and controls. There were six and five multifragmentary (23 A3) fractures in cases and controls. The distribution of this was not significantly different between the groups. The demographic variables are summarised in Table I. Both the groups were matched to demographic variables and radiograph parameters of volar tilt, radial inclination, and radial length before M&R. There was no difference between the groups on radiological parameters. The volar tilt did demonstrate a significant difference between both the groups on the difference in difference assessments (P=0.05); however, the other radiograph indices did not. A summary of primary and secondary (radiographic) outcomes is presented in Table II.

**Table I:** Demographic variables between group I and II.

Parameter	Group I (n=39) cases	Group II (n=40) controls	P value
Mean age in years ±SD	40.42±19.08	45.47±14.96	0.20
Gender Male/Female	19/20	27/13	0.09
Mechanism of Injury 1 A/B/C/D/E	16/18/2/3/0	13/19/0/4/4	0.17
Side Fractured Left/Right	23/16	16/24	0.08
Dorsal Comminution	7	7	0.81
Volar tilt	5	1	0.19
Dorsal Tilt in degrees	13.61±14.66 (CI 9.00-18.21)	16.40±12.78 (CI 12.44-20.36)	0.29
Radial height in mm	6.69±4.03 (5.42-7.95)	6.84±3.16 (5.86-7.81)	0.84
Radial inclination in degrees	18.18±7.46 (15.83-20.52)	16.95±8.97 (14.17-19.73)	0.44

Notes – 1: (A) slip and FOOSH, (B) road traffic accident, (C) fall from height, (D) sports related injury, (E) others (physical assault)

**Table II:** Radiographic outcomes.

Parameter	Time of evaluation	Group I (Cases) N=39 Mean±SD (CI)	Group II (Controls) N=40	Difference	P value
Volar Tilt in degrees	Pre-reduction	13.61±14.66 (9.07 to 18.1)	16.40±12.78 (12.4 to 20.4)	-2.785	0.29
	Post-reduction	6.76±7.39 (4.44-9.07)	2.35±10.35 (-0.85-5.55)	- 4.413	0.10
		Difference in difference		7.198	0.05
Radial height in mm	Pre-reduction	6.69±4.03 (5.44 to 7.94)	6.84±3.16 (5.86 to 7.82)	-0.152	0.84
	Post-reduction	10.03±3.33 (8.98-11.07)	8.77±3.11 (7.80-9.73)	1.263	0.10
		Difference in difference		1.41	0.20
Radial inclination in degrees	Pre-reduction	18.18±7.46 (15.9 to 20.5)	16.95±8.97 (14.2 to 19.7)	1.232	0.44
	Post-reduction	21.12±3.4 (20.05-22.18)	19.37±6.83 (17.25-21.48)	1.753	0.27
		Difference in difference		0.521	0.82

We had an unacceptable reduction of 9 (22%) and 18 (46%) (P=0.70) DRF between groups I and II after M&R as per the set reference. We had 1, 4 (Radial height), 2, 4 (radial inclination), and 6, 10 (volar tilt) unacceptable reductions in groups I and II as per the set reference. We estimated a reduction in the incidence of mal-reduction by 49% (Risk ratio 0.51) and an absolute risk reduction of 22% through USG-guided reduction. We estimated a number needed to treat four through USG-directed M&R of DRF to prevent one unacceptable reduction.

## Discussion

This trial aimed to answer the accuracy of Ultrasound-guided closed reduction in DRF on radiographs. We hypothesised that ultrasound-guided manipulation would improve the radiograph indices compared to the conventional technique. However, it did not reflect in our outcomes. The volar tilt improved on USG-directed manipulation on assessments of difference in difference. Two randomised controlled trials directly compared USG-guided reduction to conventional closed reductions in DRF and found no difference in the adequacy of reduction, re-manipulation rate, time to reduction, and need for operative intervention^7,8^. One of these trials was inadequately powered. Both the trials did not define the fracture type for inclusion criteria. Both trials did not report the mean correction in the radiograph parameters achieved by USG-guided M&R. We believe that the subcutaneous location of bone facilitated easy palpation and reduction responsible for observed outcomes. An RCT ascribed clinician experience as a reason for no differences in USG guided and blind M&R; however, we had orthopaedic residents (1-3 years’ experience) who did M&R for the participants of this study. Though M&R by conventional technique did manifest as more failures compared to USG-directed reduction, it did not reflect a significantly different comparison between the groups. We observe that USG as an adjunct to reduction visualised cortical alignment for two planes in all the studies for adequacy of reduction. However, it does not translate to the correction of radiograph indices. It could explain the reason for the outcomes of this and other trials. The presence of fracture comminution and impaction at the fracture site could be a confounder for judging the adequacy of closed reduction across all studies on USG.

Evaluation of radiographic parameters after the first attempt of M&R was the endpoint of our trial. Therefore, we could not compare the radiographic outcomes of our trial with other published studies^6-9^. However, we looked at the rates of unacceptable reduction, operative intervention, and the need to repeat manipulation in published series^6-9^. We found a cumulative rate ranging from 6.5% to 16.9% in the USG and 26% to 37% in conventional M&R^6,9^. Two studies directly reported a significant difference in volar tilt favouring the USG-directed M&R; however, the design of these studies was case-control and ambispective^6,9^. One study compared the cost incurred between radiographic (USG and Fluoroscopy) and conventional landmark-guided reductions in DRF and found the former costlier.

Low cost, repeatability, and free from the effects of radiation were the strengths of the use of ultrasound-directed M&R. It offered a point-of-care solution for the treatment of DRF in this and several other studies; however, it is reader-dependent, which could have introduced significant interobserver variability in assessments at M&R. therefore, we believe it is unlikely to change the current practice of blind anatomic landmark guided M&R in DRF. We recommend a trial comparing sonographic-directed DRF M&R and functional evaluation because several studies have demonstrated better functional outcomes after correcting radiographic indices.

Limitations of the study, to match patient-specific comparisons, we could have compared the fractured distal radius radiographic parameters to the contralateral side. However, ethical permissions to image the normal side limited our plans.

## Conclusion

The conventional method for reducing Colle’s fracture based on palpation of anatomical landmarks does not benefit from the addition of USG-guided imaging; however, better volar tilt measurements translate to functional improvements that need further investigation.
